# Technical and economic design of a novel hybrid system photovoltaic/wind/hydrokinetic to supply a group of sustainable buildings in the shape of airplanes

**DOI:** 10.1016/j.heliyon.2023.e14137

**Published:** 2023-03-05

**Authors:** Daniel Icaza, David Borge-Diez

**Affiliations:** a Postgraduate Unit, Catholic University of Cuenca, Ecuador; b University of Leon, Campus of Vegazana S/N, Spain; c Department of Electrical, Systems and Automation Engineering. University of León, Spain

**Keywords:** Renewable energy, Techno-economic analysis, Energy, Hybrid system, Sustainable buildings

## Abstract

This research analyzes the impact of a hybrid off-grid renewable energy system consisting of wind turbines, solar photovoltaic, hydrokinetic turbines and battery-backed to provide a group of novel airplane-shaped buildings, generates development in nearby towns that sit on a city vantage point from Cuenca in Ecuador. This is an innovative proposal that, in addition to using renewable energy in the complex of buildings, generates development in nearby towns. Three sources of renewable energy under energy control, load cycle and load monitoring are used to determine new patterns in the behavior of the sources with respect to the demand for electricity. Above all, it reduces carbon. With the support of HOMER Pro, the generation sources are optimized to cover the electrical demand patterns of the group of buildings in the form of airplanes. The results show that the systems that include solar panels, wind and hydrokinetic generators have a higher cost but there is more guarantee by maintaining their charge levels in the batteries above 40%. The proposed methodology and design can be widely adapted to places with similar characteristics worldwide, creating a novel solution for this type of buildings powered by renewable energy. The annual energy required by the set of buildings is 234.86 MW h/year. When projecting the renewable energy system for 25 years, an NPC of $37,600 and a COE of $0.386/kWh are achieved.

## 1 Introduction

Over the years, thermal generators have been the main source of electricity production for the five continents and oil has been the source of development in the regions. Among the largest suppliers of crude oil have been characterized the countries of the Middle East, South America and Africa, their destinations of these resources have preferred North America and Europe. The consumption of energy in high proportions has been directly linked to the large volumes of atmospheric pollution and global warming that is increasing every time [1]. The urgency of taking immediate action has led the countries of the five continents voluntarily and responsibly to attend the 2015 Paris summit called COP 21 [2]. At this summit, it was proposed to avoid the increase in world temperature to 2 °C to avoid catastrophes in some parts of the world as a result of climatic changes. In order to restrict climate increase, the need to make sufficient investments to transform polluting systems into environmentally friendly systems with low carbon emissions was raised. In this sense, renewable energies are an interesting option to replace fossil fuels and progressively reach high levels of penetration in world electricity markets [3]. Renewable energies that are very well coupled to current renewable energy systems in addition to the old hydraulic technology, are especially wind, solar, biomass, geothermal. According to the International Energy Agency (IEA) [4], it considers that the demand for electrical energy will increase by 4.6% at the beginning of 2022, supported by the recovery of economic activity.

Domenico Mazzeo et al. [5] carried out a World Geographic Mapping and presented the optimization of the technical-economic performance of hybrid stand-alone and grid-connected renewable systems in all Köppen-Geiger climates. Then in his new research [6] he used a method with energy reliability constraints for the multi-objective optimization of a hybrid photovoltaic-wind system with battery storage. In recent years, great advances have been made in renewable energy systems, so reference [7] carried out a solid review of the literature and a statistical analysis of hybrid photovoltaic-wind renewable energy systems, for which it used 550 articles most relevant. Reference [8] presented an innovative research to carry out multi-criteria energy-economic-environmental decision-making in the optimization of a hybrid renewable system.

In this same line of development, H.R.Baghaee et al. [9] presented the cost-based multi-objective Pareto optimal design and reliability study of an autonomous generation microgrid system. R. Luna-Rubio et al. [10] presented a new methodology after conducting an exhaustive review of optimal sizing of renewable hybrid energy systems. Makbul A. M. Ramli et al. [11] presented a techno-economic study of the wind/solar hybrid system for the western coastal area of Saudi Arabia. All these studies determine that the best technical and economic options are hybrid systems and they best complement each other. It is also determined that renewable energies are the most viable path in the fight against climate change and greater investments in these technologies are desired.

Two decades ago, renewable energies had quite high production costs which made it impossible to have isolated equipment or connected to the network, the commercialization was still restricted [12]. In recent years, the equipment for the use of renewable energy has had a substantial drop in its sale prices to the public, which allows the different countries to obtain new facilities for the production of renewable energy [13]. Nowadays, in addition to having these technologies with more accessible prices, greater confidence has been generated in carrying out these installations [14]. Above all, the penetration levels of wind and solar photovoltaic have grown exponentially worldwide [15].

In this article, the existing renewable resources are used in a study site near the City of Cuenca, such as wind, solar radiation and water current. These resources are optimized with the purpose of avoiding exhaustive investments and, above all, allowing this group of buildings to be economically viable. In reality, it is a fantastic study that will allow to be built in different places of Ecuador in accordance with the current Ecuadorian National Development Plan (ENDP) 2021–2030 and may very well be taken as a reference in other parts of the world. The essence of this study is to have primary energy resources on site that allow them to be transformed into electrical energy and contrast with their landscapes to create places of rest and recreation. Another element that is sought to take advantage of is the particular characteristics such as the view of the city and promote the economic development of the localities in harmony with the environment.

## 2 Literature review

Solar and wind power reached 67% of new added electric power capacity globally in 2019 [16]. The growing level of confidence that renewable energy sources have generated among citizens has been the product of a slow process that is just beginning to take shape in different countries. The benefits have been substantially identified in rural areas [17]. The implementation of these renewable energy systems are not complex and mainly in sectors that historically did not have access to electricity, currently they can be developed with new companies, better land use and add value by generating new jobs [18]. Another compelling reason is that in locations far from urban centers, fuels are excessively expensive due to the need for transportation to finally store them in specific places that on many occasions have even generated accidents due to the lack of sufficient care and safety [19].

Different researchers addressed the importance of forming hybrid energy systems, taking advantage of the geographical conditions and the energy potential of the localities. By having open water channels, it is possible to take advantage of their energy capacity using small turbines and together with other generation systems it is possible to form hybrid systems. Knowing that the different energy sources are intermittent, different researchers have proposed including storage systems for times of high demand. However, before making investments it is necessary to be sure that it is techno-economically feasible to satisfy the electricity demand. In this sense, the research is combined in the modeling of hybrid systems, the simulation and their dimensioning [20].

Sandra T. Matarneh et al. [21] present Building Information Modeling for novel facility management and predict that research in this trace field will take several innovative directions. A Farzaneh et al. [21] after his review of the building literature, he considers that new energy models of buildings are required during the design process to better integrate electrical installations into buildings. L Belussi et al. [22] insist that it is essential to design new autonomous energy systems that are consistent with the performance postulates of zero energy buildings.

Mohammad Hossein Jahangir [23] analyzed the benefit that various renewable energy resources available in port areas can provide and his simulation results prove to be feasible and lead to implementations in other places even with different climates.

Sonja Kallio and Monica Siroux [24] presented the characteristic designs of hybrid renewable energy systems. The study found that an important solution to overcome the typical fluctuation of several renewable energy inputs, including wind and photovoltaic energy, is overcome with controllable sources, such as biomass. In this regard, Xiaofeng Zhang et al. [25] considers it necessary to fully optimize hybrid systems with energy storage and analyzes the thermal/hydrogen combination based on biomass for buildings and hydrogen vehicles.

There are different methodologies that have been adopted in different investigations in recent times, among them the following stand out: Jijian Lian et al. [26] in its analysis uses the methodology that solves the problems of variability of most renewable energy sources, considering one of them as the basis of the other sources with less variability. The study suggests develop hybrid systems that include hydroelectric power or hydroelectric energy storage to increase stability and guarantee continuity of electricity service.

Hemant Sharma et al. [27] in his study makes a comparison of evaluation methodologies with simulation software for hybrid energy systems. The development in the last 10 years of specialized tool such as Homer Pro, Energy Pro, iHOGA and TRNSYS to analyze these systems stands out. The research compares these different tools in detail, in terms of environmental and economic evaluation. Concluded that only one integrates techno-economic impacts.

From this it follows that the software is very useful for conducting analyzes in different parts of the world. Fabian Eze et al. [28] conducted an assessment of the economic and technical feasibility of hybrid renewable energies in the Kenya Institutional Building. The total net current cost over 25 years was calculated to determine the lowest energy cost for the Photovoltaic/Diesel/Grid and the optimal design of the system was found.

On the other hand, there is currently a significant concern about housing. In Ecuador it is argued that housing is a right of every citizen, to which it is added that environmental sustainability must be promoted. In this regard, there is a growing development in the integration of sustainable housing and electrical energy systems through renewable sources.

**Fig. 1:**
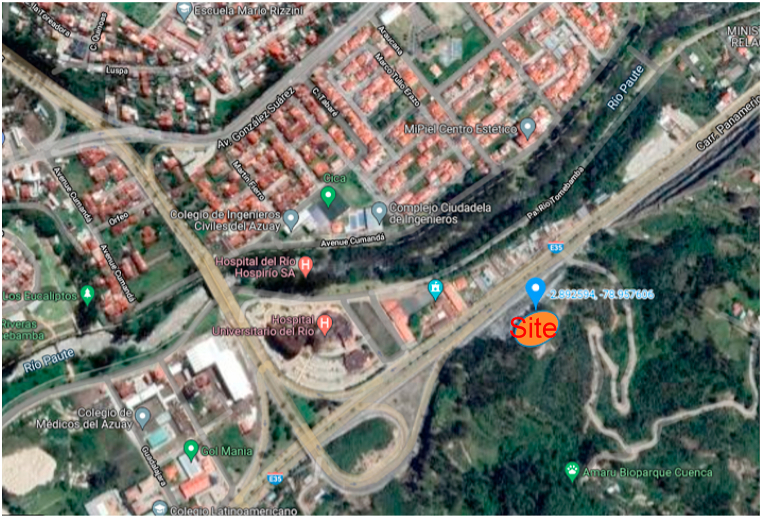
Location of the site under study overlooking the City of Cuenca in Ecuador.

The city of Cuenca has attractive places around the city and specifically the northeast area has a special vegetation. In this regard, concrete actions are developed, which includes a network of novel airplane-type buildings with views of the city and that contrast with nature. Its interiors have great features and basic services, which includes electrical service. In this sense, the focus of this research is to take advantage of the characteristics available on site for the supply of electrical energy through renewable energy sources. These include the hydrokinetics of the Paute River that is at the foot of the town where this study is carried out, the use of the wind from the mountain range and the photovoltaic solar energy available on the site. According to the Köppen-Geiger climate classification [29], the City of Cuenca has a climate of *As*, which corresponds to a dry summer equatorial. The site under analysis is located in the lower part of the Amaru Biopark in the City of Cuenca with the coordinates (−2.892594, −79.957606) as shown in Fig. 1.

**Fig. 2:**
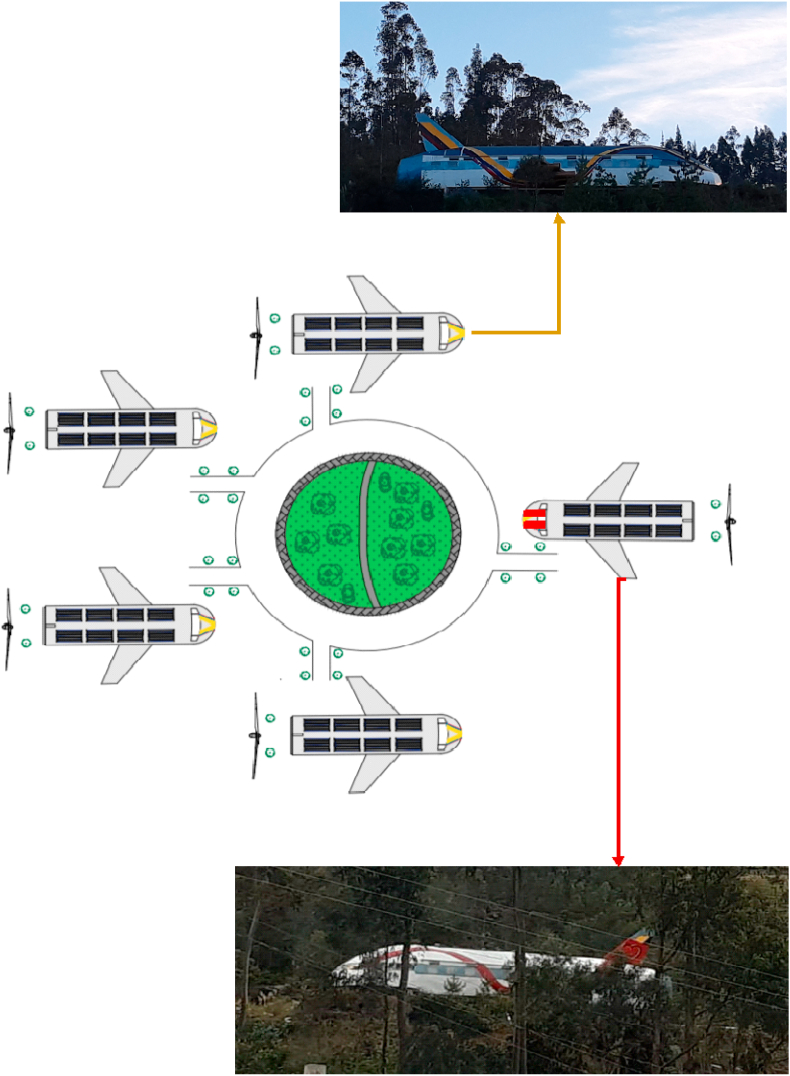
Configuration of the group of sustainable buildings.

In Fig. 2 is shown see the configuration of the five buildings that are located four in a south direction with colors of the Ecuadorian flag (Yellow, blue and red) and one in a north direction with colors of the local flag (yellow, white and red). All these airplane-type buildings have the same interior characteristics, they are 30 m long and 12 m wide. They are new cozy buildings, have very comfortable interior spaces and are exceptionally attractive. Initially, this project started in the town of El Valle in the Cuenca Canton itself and the only source of study was through the use of photovoltaic solar energy [30] for a single building. After this experience, it is considered that the best option is to have a novel group of buildings due to the high demand of guests. It was considered to electrify these buildings using renewable energy and without connection to the public electricity grid.

**Fig. 3:**
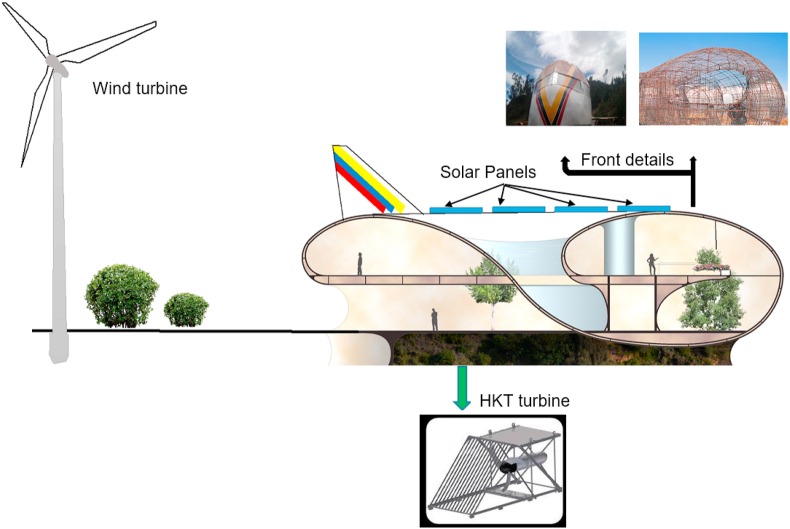
Hybrid system detail.

In Fig. 3 you can see a section of the sustainable building and its internal forms practically without edges, they are peculiar spaces where natural lighting will be used to the maximum during the day, therefore the position of each building is important, they are all the buildings on the side. At necessary times and at night hours, the energy generated is available to power lighting circuits and electrical outlets. To heat water and cook, Liquefied Petroleum Gas (LPG) is used, which is subsidized by the Ecuadorian state [31].

From these spaces you can enjoy the exceptional panorama of the city of Cuenca and the colonial architecture that is valued by UNESCO as a Cultural Heritage of humanity [31]. The energy generated is provided by the hybrid system to electrify each building. The exceptional conditions of the locality such as wind speed, solar radiation and the kinetics of the water of the Paute River are considered [32].

The paper is organized as follows: In the first section there is the introduction, in section 2 a thorough review of the state of the art is carried out and the methodology is presented, in section 3 the hybrid system model is optimized and the parameters are analyzed. main. In section 4 the technoeconomic analysis is carried out, in section 5 the respective results are presented and discussed to finally issue the conclusions.

### 2.1 Methodology

**Fig. 4:**
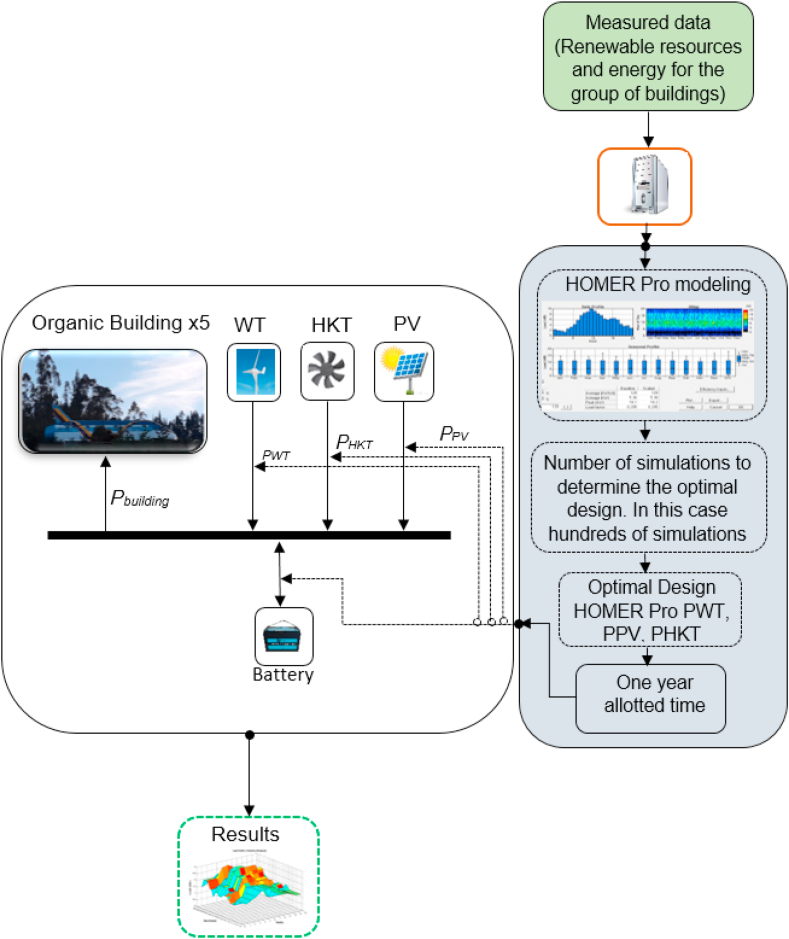
Requested scheme for the sizing of renewable sources.

The methodology used for the configuration of the hybrid electricity generation system for the group of airplane-type buildings located on the outskirts of the City of Cuenca is based on potentially favorable meteorological data related to the site. To carry out this study in a unified way, the Homer Pro tool is used, it is a widely used software due to its versatility, it has an information base of existing equipment on the market and allows simulations under different conditions. It also allows to establish a direct link with matlab, interacting effectively and establishing the best options for the hybrid system's operation. These tools also allow an economic analysis in relation to energy production levels. The hybrid system study methodology that includes the economic analysis is presented schematically in Fig. 4.

The renewable energy sources are composed of wind, solar photovoltaic and hydrokinetic energy that will work according to the resources available in the city of Cuenca. The storage system is also integrated, it is composed of a general battery bank to supply the 5 buildings contemplated. The generation sources are connected to DC bus. Only the hidrokinetic turbine is connected to the AC bus and requires an inverter. Ancillary services must be provided at all times.

This research becomes novel by extracting the renewable resources available on the site in a unified environment between the building, clean energy sources and landscape environments. The software used manages to evaluate the useable resource in accordance with the demand of the group of airplane-type buildings.

Homer Pro is a versatile software for simulating electrical production systems in different conditions and locations with a platform that includes a variety of energy sources. The most important thing is that Homer allows you to carry out both technical and financial analyzes in a flexible way, which makes a noticeable difference with the rest of the simulation tools for hybrid systems that focus on the eminently technical. In addition, it allows the use of its libraries with a diversity of energy sources to achieve combinations according to availability and design challenges. Also, the libraries that are available for simulation adhere to reality since they are equipment that are available in the market and not only have the purpose of conducting research studies but also that they are implemented. It is a software that has a free access time for 21 days and then will require a license, it has several modules according to the requirements, among them are: simulation module, sensitivity and optimization analysis.

## 3 Optimization

**Fig. 5:**
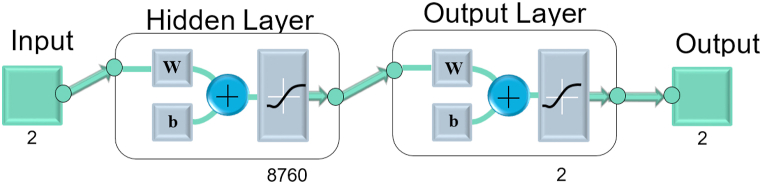
Basic structure of ANN techniques.

**Fig. 6:**
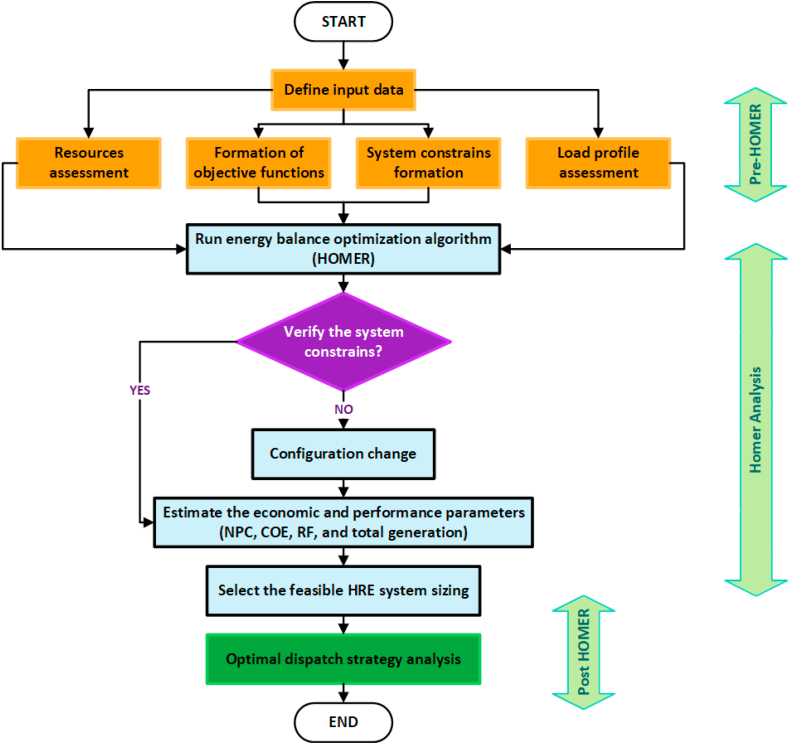
Flow diagram of the studied system.

The purpose of this research is to design an energy system that guarantees quality and continuous electrical service for sustainable airplane-type buildings overlooking the city of Cuenca, it is a novel approach. Based on meteorological data related to the WT, PV and HKT sources, the aim is to properly take advantage of renewable energy resources and supply the total load demand according to historical data through the Artificial Neural Network (ANN) technique. The optimal size is also defined with the shipping strategies of the Historic Renewable Energy (HRE) configuration to be analyzed to achieve the economic and ecologically sustainable returns. The proposal includes an analysis using the basic structure of the ANN techniques presented in Fig. 5, the optimization diagram shown in Fig. 6 is also included.

The optimization process flow was developed according to the requirements of the buildings under study, based on the literature related to hybrid renewable energy systems.

### 3.1 Renewable energy system modeling

The renewable energy system highlighted in this study consists of a combination of PV/HKT/WT supported by battery storage units. Source by source of generation and its equipment are analyzed below.

#### 3.1.1 Photovoltaic generator

These buildings with special characteristics in the shape of a plane consider solar power as the reference source within the configuration, given the characteristics of the site, without being excellent, they meet very good conditions to take advantage of solar radiation, in fact there are various applications in the city of Cuenca, which provides confidence to structure sustained applications in solar energy as explained in Ref. [33].

On each airplane-type building there are 8 monocrystalline-type modules, mainly to the use by 166 mm 24VDC cells, 9 bus bars, each with 230Wp.

The maximum power output from solar panels becomes a paramount parameter and is the reason for solar photovoltaic investment. The output power of the panel is determined by equation (1):

(1) $$P_{pv}=f_{pv}*\;Y_{pv}*\;\frac{I_{T}}{I_{S}}$$

Where $Y_{PV}$ is the nominal power of the panels, $I_{T}$ is the radiation on the solar panel (kWh/m^2^), $I_{S}$ is usually used as a reference value of 1000 W/m^2^ assumed as an optimum irradiation value, $f_{PV}$ is the loss parameter, such as distance from electricity conductors, due to joints between conductors, increased dust and dirt on the surface of the solar panel, variations in ambient temperature that influence the performance of the panel.

The more stable the ambient temperature, the better it will be, it can be seen that the operational part itself, which depends on solar radiation, is added to the part. It can be seen in equation (2):

(2) $$T_{C}=T_{amb}+G_{S}\;\left(\frac{NOCT-25}{1000}\right)$$

Where $T_{amb}$ is considered the ambient temperature, $G_{S}$ represents the global solar irradiation in the site, while NOCT corresponds to the characterized temperature of the solar cell.

#### 3.1.2 HKT turbine

This research includes a small hydrokinetic turbine, a 380 kg smart monofloat model, which operates between 90 and 230 rpm and provides a maximum electrical power of 5 kW. It is feasible to open small channels bordering the river taking advantage of the water flows coming from the mountainous heights that cross the city of Cuenca and that unleash in the Paute river.

The selected turbine operates at 0.6 bar pressure, the hydraulic pressure at its maximum working point is 4 kg/cm2. It is a relatively cheap equipment compared to small size turbines. They are very friendly to the environment, nor do they produce landscaping alterations since the equipment is aligned with the duct. It has a 2″ diameter at the entrance and the integrity of the equipment is not put at risk due to the high rainfall that occurs in the area at certain times of the year. The supplied voltage is 24 V in direct current.

The power at the terminals in the hydrokinetic turbine $P_{HKT}$ is expressed as a function of time by Eq. (3). $\rho_{W}$ is the density of the water in kg/m³, A is the area of the hydrokinetic turbine given in m^2^, ($C_{p,h}$) is the power coefficient, $\eta_{HKT}$ is the performance of the turbine, the speed of the river is *v* in *m/s* measured at the point of installation of the turbine.

(3) $$P_{HKT}=\frac{1}{2}*\;\rho_{W}*A*v^{3}*C_{p,h}*\;\eta_{HKT}*t$$

The real power produced in the 8760 h of the year that the turbine must be operating is obtained by means of Eq. (4):

(4) $$E_{HKT}=\sum_{t=1}^{t=8760h}P_{HKT}\left(t\right)*t$$

#### 3.1.3 Wind turbine

The power P_WT_ generated by the wind machine can be defined using the mathematical relationship (Equation (5) which mainly depends on wind input speed (*v*_*W*_), $\rho_{a}$ is air density, *C*_*p*_ is Betz coefficient is in function angle *β* and *λ*. The relation *λ* defined as λ = *Rω*_*m*_*/v*, *R* is the turbine radius, *ω*_*m*_ is the velocity angle of the turbine shaft*.*

(5) $$P_{WT}\left(t\right)=\frac{1}{2}C_{p}\left(\lambda ,\beta \right)\rho_{a}{Av_{w}}^{3}$$

The power delivered by the wind turbine is a function of the wind speed, is considered by limited operating ranges expressed by Equation (6), analyzed by Ref. [34]:

To generate energy, the wind turbine requires directly on the wind speed in 2 regions.

1) Turbine operation with a speed higher than the rated wind speed *v*_*r*_*.*
2) Wind speed below nominal. It is experienced when the load is below the nominal power $P_{WT}^{ab}$. To obtain constant power at the terminals of the electric generator, speeds higher than the nominal value are required. By having wind speeds that exceed the nominal value, the turbine delivers constant power. If there is a very excessive speed that exceeds 20 m/s, the wind turbine stops for safety. As a reference, the minimum speed is *v*_*i*_ and the cutting speed is *v*_*c*_*.*

(6) $$P_{WT}^{av}\left(t\right)=\left\{ \begin{array}{c} 0\;if\;v_{w}<\;v_{i}\\ \frac{1}{2}C_{p}\left(\lambda ,\beta \right)\rho_{a}{Av}^{3}\left(t\right)\;if\;V_{i}\leq v_{w}\leq v_{r}\\ {\;P}_{w}^{r}\;if\;v_{r}<v_{w}<\;v_{c}\\ 0\;if\;v_{w}>\;v_{c} \end{array}\right.$$

#### 3.1.4 Batteries

The surplus energy is commonly stored in a battery bank, used mainly at night, as there is greater consumption of lighting systems and outlets inside buildings.

The design includes 5 battery storage blocks, which cover the joint demand between buildings. Due to their special characteristics, these batteries have a useful life of 10 years, essential to maintain the continuity of the electric power service inside these special buildings. Users seek to have all the services and enjoy moments of tranquility, relaxation and take unforgettable moments with them.

To calculate the number of batteries $\left(N_{bat}\right)$ the useful life of the hybrid system is determined for 20 years, this implies that there must be a replacement after 10 years with special and well-maintained batteries, it is calculated through the mathematical relationship (7).

(7) $$N_{bat}=Cell\left(\frac{{Life}_{HS}\;*\;{Life}_{bt}^{pu,year}}{T_{bat}^{life}}\right)$$

${Life}_{HS}$: Factor that evaluates the life cycle of the renewable energy system.
$T_{bat}^{life}$: Time in years prior to battery bank replacement.
${Life}_{bt}^{pu,year}$: Battery charge level in the last year.

According to the levels of energy generation produced by the three renewable sources and the electrical power required by the demand of the set of aircraft-type buildings, the energy is also stored in a battery bank. The State of Charge (SOC) is related to discharge levels. It is determined by Equation (8) and Equation (9) provided by Ref. [35].

(8) $$SOC\left(T\right)=SOC\left(t-1\right)+\frac{E_{bat}\left(t\right)*\;\eta_{cbat}}{P_{bat}}*100$$

(9) $$SOC\left(T\right)=SOC\left(t-1\right)+\frac{E_{bat}\left(t\right)*\;\eta_{dbat}}{P_{bat}}*100$$

$E_{bat}$: Battery capacity with charge and discharge $\eta_{cbat}$ and $\eta_{dbat}$ efficiencies.

Another reason for the battery bank in addition to storing energy is to maintain the balance between energy supply and demand. The power balance is evaluated over time using the following mathematical relationships.

• $P_{PV}^{T}+P_{HKT}^{T}+P_{WT}^{T}=P_{DEMAND}^{T}$ It maintains a complete balance.
• $P_{PV}^{T}+P_{HKT}^{T}+P_{WT}^{T}>P_{DEMAND}^{T}$ The hybrid system generates more electrical power than is necessary at the extreme of demand. The battery bank maintains its full charge over time and is expressed by equation 10

(10) $$E_{bat}^{T}-E_{bat}^{T-1}.\left(1-\tau \right)+\left[\left(P_{PV}^{T}+P_{HKT}^{T}+P_{WT}^{T}\right)-\frac{P_{l}^{T}}{n_{inv}}\right]\eta_{bc}$$

Where.

$E_{bat}^{T}$ and $E_{bat}^{T-1}$: Battery charge at time *T and T-1.*
$\eta_{bc}$: Battery bank efficiency.
$\eta_{inv}$: Inverter efficiency.
τ: Autonomy time.
$P_{PV}^{T}$: Electrical power provided by the solar panel.
$P_{l}^{T}$: Electrical power absorbed by the demand in a certain time.
$P_{HKT}^{T}$: Electrical power provided by HKT.
$P_{WT}^{T}$: Electrical power provided by WT.
•$P_{PV}^{T}+P_{HKT}^{T}+P_{WT}^{T}<P_{DEMAND}^{T}$ The power absorbed by the demand exceeds the levels of electrical power generation generated by the three renewable sources. In this condition, the battery provides enough electrical power stored, as identified in the mathematical relationship (11) [37].

(11) $$E_{bat}^{T}-E_{bat}^{T-1}.\left(1-\tau \right)+\left[\frac{P_{l}^{T}}{\eta_{inv}}-\left(P_{PV}^{T}+P_{HKT}^{T}+P_{WT}^{T}\right)\right]\eta_{bf}$$

$\eta_{bf}$: Performance of the energy storage system.

#### 3.1.5 Inverter

The equipment established to convert direct current into alternating current brings with it an efficiency that depends on construction factors. The mathematical relationship is given by equation (12):

(12) $$P_{o}=P_{i}*\eta_{inv}$$

The efficiency $\eta_{inv}$ is given by the division between power output $P_{o}$ and power input $P_{i}$.

$P_{i}$ is the sum of the partial powers of the solar, wind and hydrokinetic, according to analysis of references [38] and [7].

### 3.2 Total power generated

The configuration of the hybrid system will allow having a complete generation profile to supply the group of airplane-type buildings according to equation (13):

(13) $$P_{total}\left(t\right)=\sum_{pv=1}^{Sn}P_{PV}\left(t\right)+\sum_{w=1}^{Sm}P_{HKT}\left(t\right)+\sum_{WT=1}^{So}P_{WT}\left(t\right)$$

The parameter Sn corresponds to the total number of solar photovoltaic panels. Sm is the number of hydrokinetic turbines and So the wind turbines.

#### 3.2.1 Optimal design

**Fig. 7:**
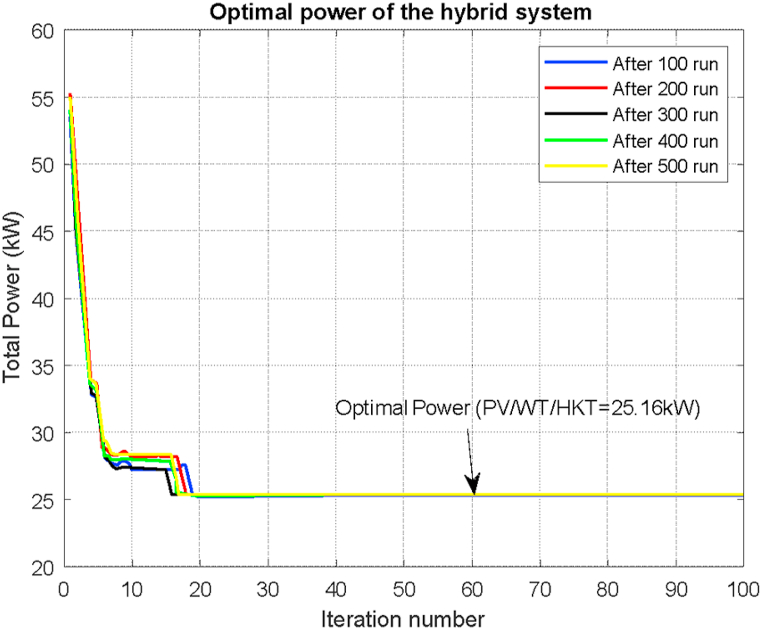
Hybrid System Optimal power responses for PV/WT/HKT.

Homer Pro is a tool that has the optimization module and after making blocks of runs in WT/HKT, PV/HKT, PV/WT, PV/WT/HKT combinations, the iterations of the latter are considered as a less fluctuating mix. and the results of the optimization process brings maximum production and less installed power. It guarantees sufficient power to the load that it will maintain throughout the day. In Fig. 7 it can be seen that the optimum power is 25.16 kW.

The decision variables of the optimization problem are specified according to (15). The objective function (to be minimized) is characterized by the sum of the different terms that are duly weighted: the deviation of the various demands (electrical and thermal) are intended to depend directly on the energy produced (when possible) and the energy of the storage system (that was maximized during the optimization horizon.

## 4 Techno-economic analysis

This study focuses specifically on the electrical system, and other aspects such as its architectural and civil structure and others are not discussed. The aim is to keep the Present Cost (NPC) to a minimum of the set of elements that make up the hybrid system widely mentioned in the previous sections. The photovoltaic solar system is essential and on this source hydrokinetics is incorporated as well as wind for optimal configuration. On the other hand, it is important to determine the Total Annual Cost (TAC), it is fundamental within the analysis of the project, it is calculated by means of equation (14):

(14) $$TAC=C_{acap}+\sum_{i=1}^{n}C_{0\&M,i}+C_{f}+\sum_{i=1}^{n}C_{R,i}$$

Contemplating a scheme of minimized costs $\mathrm{Min}\;M_{t}\left(P_{pvD}\left(t\right),P_{pvA}\left(t\right),P_{wt}\left(t\right),P_{Bat}\left(t\right),P_{hkt}\left(t\right)\right)$ each contribution seen from the point of view of its own restrictions is evaluated through equation (15):

(15) $$\mathrm{Min}\;M_{t}\left(P_{pvD}\left(t\right),P_{pvA}\left(t\right),P_{w}\left(t\right),P_{Bat}\left(t\right),P_{hkt}\left(t\right)\right)=\mathrm{Min}\left(M_{pvD}\left(t\right),M_{pvA}\left(t\right),M_{wt}\left(t\right),M_{Bat}\left(t\right),M_{hkt}\left(t\right)\right)$$

Where $M_{t}$ is the cost of the renewable system.

$M_{pvD}\left(t\right),M_{pvA}\left(t\right),M_{w}\left(t\right),M_{Bat}\left(t\right),M_{hkt}\left(t\right)$ Parameters that refer to the individual costs of each technology and its complements for the joint operation of the hybrid system. These are costs of the photovoltaic system, wind turbines, hydrokinetic turbines and batteries.

To evaluate the total costs of the electricity production system from renewable sources, it is evaluated using the NPC parameter, these are: capital cost, O&M costs, replacement of much-needed equipment throughout the life of the project. Then it is subtracted from the current value corresponding to the income to be obtained in the planned period of time. It is determined by eqs. (16), (17).

(16) $$M_{NPC}=\frac{M_{ann,Tot}}{CRF\left(i,R_{proj}\right)}$$

$M_{NPC}$ is the net current cost, $CRF\left(i,R_{proj}\right)$ corresponds to the capital recovery factor with interest rate 1%, $M_{ann,Tot}$ is the total annual cost in $/year, and $R^{th}$ is the lifetime of the project in years, Equation (17).

(17) $$CRF\left(I,N\right)=\frac{I{\left(1+I\right)}^{N}}{{\left(1+I\right)}^{N}-1}$$

Where,

*I* = Interest rate.

*N* = Number of periods (years).

The Cost of Energy (COE) is an essencial parameter to determine for a coherent planning of energy systems in the long term, the mathematical relationship is presented in Eq. (18):

(18) $$COE=\frac{M_{ann,Tot}-M_{storage}E_{thermal}}{E_{primAC}+E_{primeDC}+E_{\mathrm{def}}+E_{gridsales}}$$

### 4.1 Data entry

**Fig. 8:**
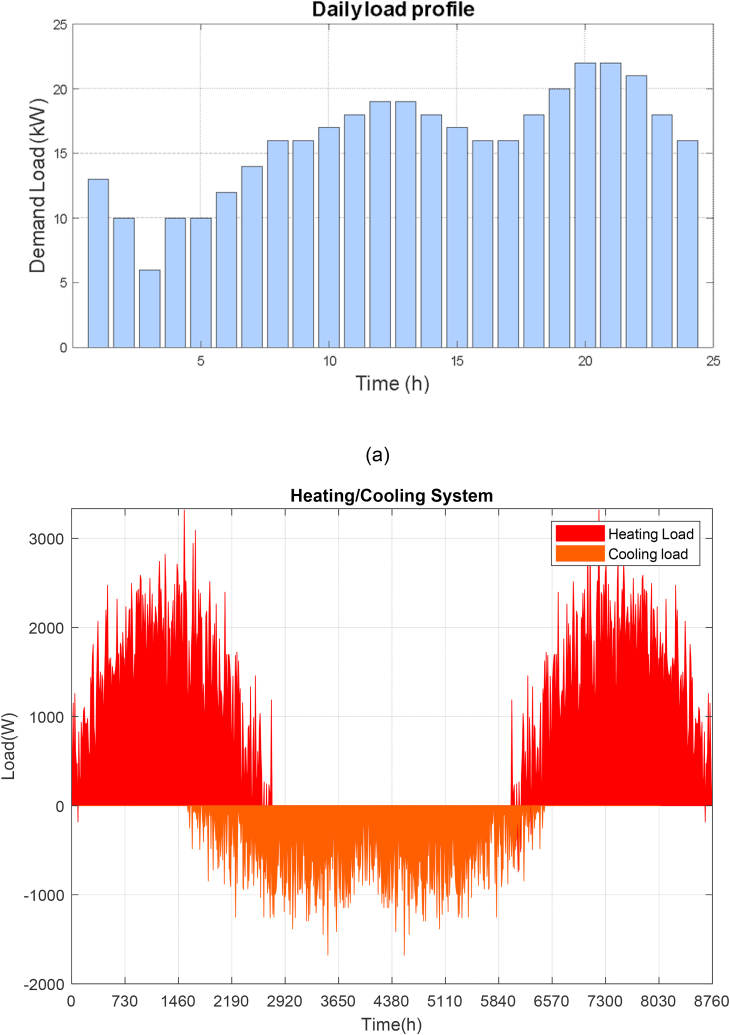
Consumption profile. (a) Daily load profile of the group of buildings under study. (b) Detail of the Cooling/Heating consumption profile.

To properly install the different equipment that makes up the hybrid system, it is important to consider the best locations on the site, especially in the position of the wind turbines, avoid shadows on the solar panels, precise location of the hydrokinetic turbine, among other aspects. All these factors will be essential and the optimal configuration will be met using the Homero Pro tool. The input parameters are essential, such as solar radiation, wind speed, water kinetics and system load. The load of the entire group of buildings under study has a consumption according to the following detail: average value (16 kW), peak value (22 kW), minimum power (6 kW) and maximum power (37 kW). Fig. 8(a) shows the normalized load demand profile for 24 h a day.

The cooling and heating loads in the buildings can be seen in Fig. 8(b). The consumption levels are not excessive, they do not exceed 3000 W of heating and 1500 W of cooling.

Heat pumps from the natural environment (air and water) will be used and transported to the interior of the buildings, heating them. It also works the other way around, taking the heat from inside the rooms to the outside, cooling them down. It is an excellent option for the present case study since it is a proven technology.

**Fig. 9:**
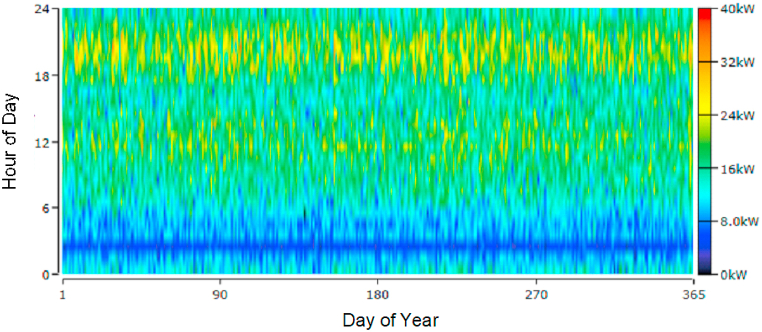
Load profile. Day of year vs Hour of day.

Homer Pro is a very versatile tool with great features. Among them, you can start from the daily load profile and obtain a behavior of the demand 365 days a year. It is possible to see the load profile as indicated in Fig. 9.

**Fig. 10:**
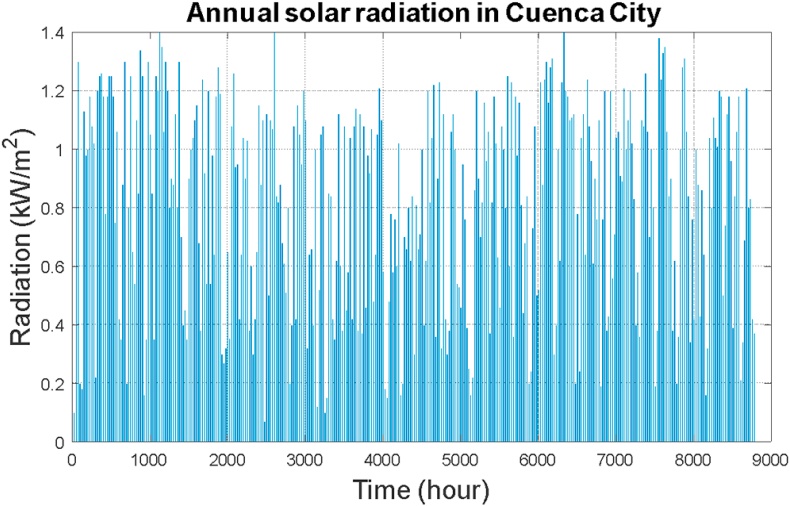
Annual solar radiation.

Ecuador continues to make progress in changes to its energy matrix, clearly showing the active participation of the national government in COP 26. There is interest, above all, in creating channels for the sale of equipment and taking advantage of photovoltaic solar energy, given the good location conditions from the equator in the middle of the world. The solar irradiation data is available in the platform for input the data profiles, it is very reliable and different researchers have placed their trust to carry out their scientific and technological developments. Fig. 10 shows the solar radiation profile for the year 2021 in the city of Cuenca. It is of the utmost importance to identify its levels since they will greatly affect the production of electrical energy from this source considered as a base to be complemented with the other two such as wind and hydrokinetic. In January there are very good levels of solar radiation and its values reach up to 1250 W/m^2^, the lowest solar radiation is commonly found in the month of July with 300 W/m^2^.

**Fig. 11:**
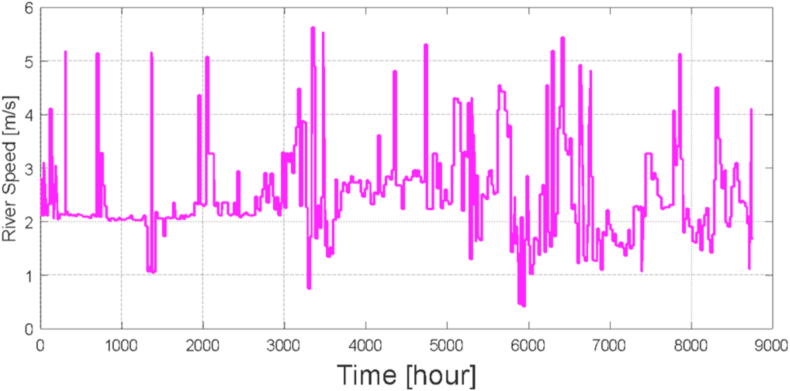
Paute River speed in m/s.

The city of Cuenca is crossed by four rivers Tomebamba, Tarqui, Yanuncay and Machángara and each one of them contributes to the Paute River. In the present study, the speed of the water is used for the purpose of generating electrical energy under socio-environmental criteria and care for the environment. Fig. 11 shows the speed profile of the Paute River at the elevation close to the specific site for the location of the hybrid system and to supply the group of special aircraft-type buildings. The average speed value of the river at the shore is 2.68 m/s.

In addition to designing a totally environmentally friendly system based on renewable energy sources, it also contemplates optimizing the equipment and power parameters that guarantee the electrical service to the group of special buildings. Among the main aspects that were addressed and are essential is the profile of the demand. The excess electrical power generated will feed ornamental external lighting circuits for greater comfort of the guests.

### 4.2 Equipment selection

To determine the appropriate equipment, its quality and recognition in the renewable energy market are considered, as well as the certainty of satisfying the demand of the entire group of sustainable buildings. Having specialized software is an important support to carry out a comprehensive evaluation from a technical as well as an economic point of view that will determine whether or not to keep the project firm. Through the application of simulations in different conditions, it is possible to identify the best operational and economic options of the hybrid system, which include equipment available in the market. The use of computer tools are of transcendental importance when designing different applications. Each configuration of equipment and the energy combinations that they offer in the hours of the day and the effective performance of the backup system to satisfy the necessary power to the set of buildings are identified. In fact, the definition of the equipment, although it is done technically and economically, ends when the investor fully accepts its results in accordance with the expectations of the study and the social approach that is intended. This is where the energy system becomes key for these renewable energy infrastructures to solve long-term electricity needs.

The configuration that is fully adequate to the objective of this study is summarized in Table 1. The essential electrical equipment and their electrical operation parameters are specified for economic valuation in order to subsequently evaluate the COE and NPC.

**Table 1:** Total components for the group of airplane-type building.

Equipment for the group aircraft-type building	Nominal power (kW)	Price per unit (dollars)	Total price
Solar panel	50	150	6000
Battery	40	400	16,000
Regulator	5 × 1	4500	22,500
HKTgenerator	5.5	3500	3500
Inverter	5 × 25	800	4000
Wind turbine	5 × 1.5	1250	6250
		Subtotal	58,250
		Volume discount	20,650
		Total (dollars)	37,600

Advantageously, the same tool allows making the respective runs to identify the optimal solution for the hybrid system with real components available on the market, as specified in Table 1.

## 5 Results and analysis

**Fig. 12:**
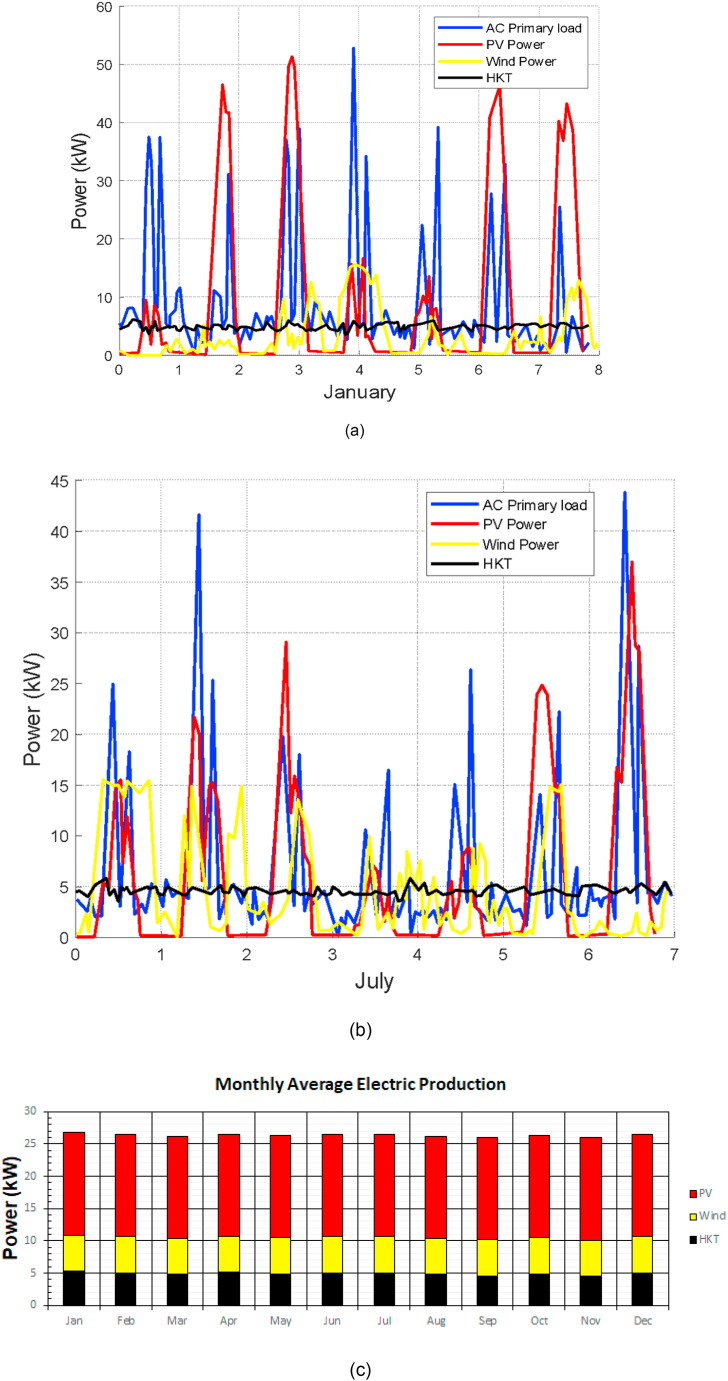
(a) Power balance for the second week of January. (b) Power balance for the second week of July. (c) Monthly Average Electric Production.

The seasonal power balance per source (PV, HKT and WT) is presented in Fig. 12, it is managed in reference to the load. It is such that the optimum power demand is compared hour by hour with the power output of the hybrid system. In the balance it includes all the power generated and the load demand curve. The power profile is shown in two months of the year, January and July, months typically studied in different simulation tools and in this case, it is no exception. Below in Fig. 12 (a) and Fig 12 (b) the energy balance is shown in each case. In Fig. 12 (c) the annual energy production profile is identified where the contribution of photovoltaic solar energy predominates.

The energy use of the photovoltaic solar system becomes substantial. The first studies related to this type of buildings arose on the basis of the use of solar energy as a single source in other locations such as the one carried out in the same Cuenca Canton in Ecuador [36] and in the City of Arequipa in Peru [37]. Thanks to the privileges that the place offers in relation to other potential renewable sources such as wind and hydrokinetic water, it allows the design of a hybrid system. This will provide energy security in the group of buildings and reduce fluctuation levels by combining sources, in addition to having a battery bank. The energy provided by HKT may not be very significant in the general energy production, however its continuity in production is of great value, in the same way if the wind source is included, the level of energy production becomes more attractive and above all everything will have a permanent generation that will allow maintaining good levels of energy storage in the batteries. Low consumption during the first 18 h of the day is a common pattern within these special buildings, creating a relatively high excess of 41.3% (4455.7 kW h/year) of the total fully renewable energy.

**Fig. 13:**
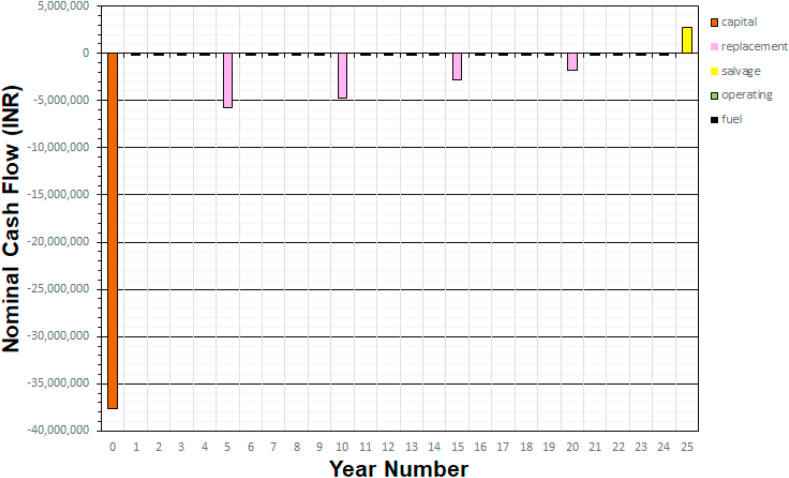
Cash flow of components.

In the Homer Pro, it is feasible to evaluate the nominal cash flow included in the different pieces of equipment that make up the hybrid system on a new screen. The representation is made by means of bars, with the downward bars being output or investment items, while the upward ones represent income. Under this scheme in Fig. 13 the first bar down represents the initial cost of the hybrid system located in the base year. As indicated, it corresponds to a negative value of money outflow, in this way there are also outflows of money for operation and maintenance and replacement. The project is proposed for 25 years, involving economic expenditures until that date. However, there is a particularity that in year 25 a positive amount arises that can be interpreted as a first income seen from the point of view of the electrical system.

In the base year, the total cost of the equipment is ($37,600). The amount invested will not allow a return of capital in a short time. The COE that the client ends up paying through an integrated invoice for the services received in the buildings does not allow a quick recovery of the capital in a short time. The development of these renewable energy systems in accordance with the current National Plan for the Creation of Opportunities 2021–2025 (NPCO), framed in Objective 5, which specifies: Promote productivity and competitiveness for sustainable economic growth in a redistributive and supportive manner, and is supported by regulation 003–11 that frames the electric power public service called: Methodological determination for the calculation of the term and the reference prices of generation and self-generation.

It is important to indicate that in Ecuador this type of project is being promoted the participation of renewable sources and within the goals of the NPCO it is to go from 65% to 90% of electrical energy with renewable sources. This project is part of those national objectives to be achieved.

**Fig. 14:**
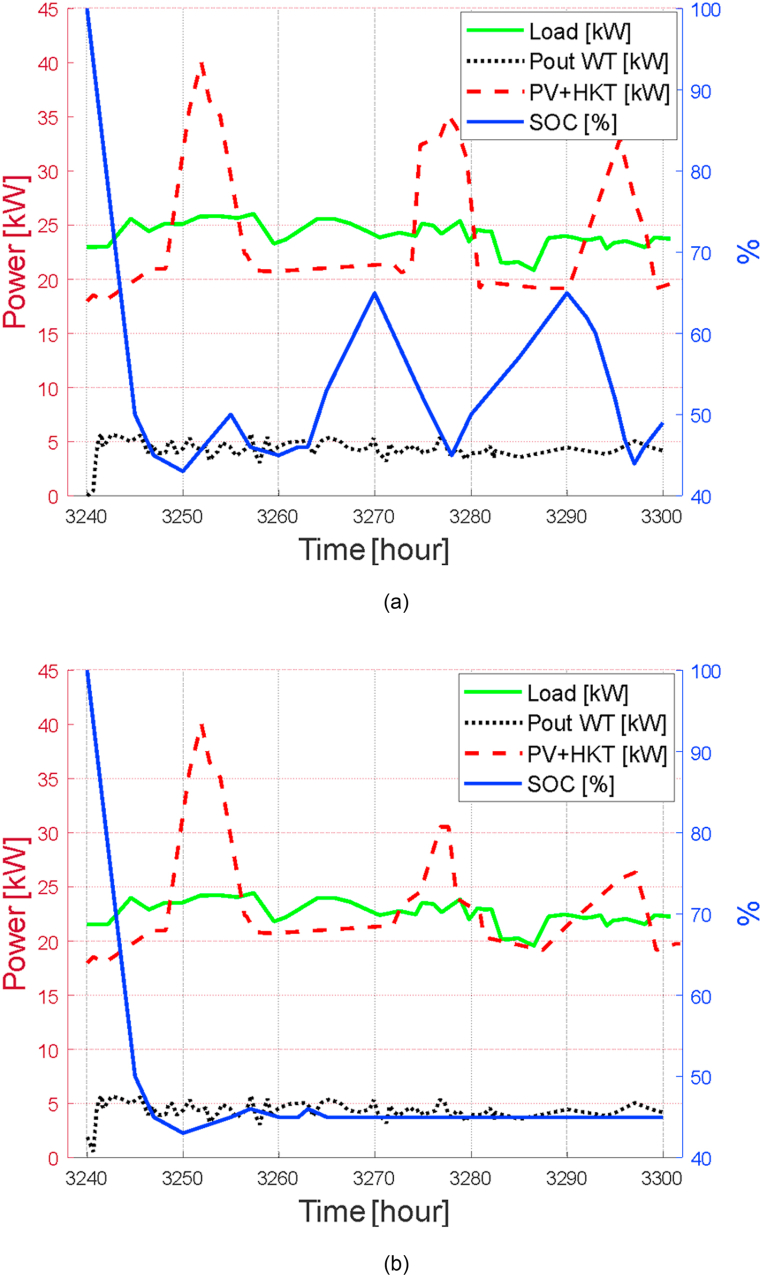
(a) LC dispatch strategy. (b) LF dispatch strategy.

Fig. 14(a) presents the electrical power balance after the renewable energy system has been simulated, including energy storage in the battery bank. In these runs, the dispatch strategy per Load Cycle (LC) has been established. It can be seen at 3256 h, the power provided by the photovoltaic and hydrokinetic system fails to provide enough power to the load comprised of the set of 5 aircraft-type buildings. In this scenario it is presented as a critical state for customers. This is when the wind system is thus with a lower proportion of power but enough to cover that deficit that the other two contributions have not achieved. It becomes interesting to analyze the whole system and the three sources become necessary, although each one of them has its own variability, but they complement each other well at different times of the day. On the other hand, when analyzing the generation scheme, a storage system becomes essential and it is precisely here that the continuity of the electrical energy service can begin to be guaranteed given any unforeseen event in one or another generation source. The storage system will allow maintaining important percentages of energy for certain hours and avoiding unforeseen events on the part of people who require permanent electrical service. It is important to clarify that natural gas is planned to be used for cooking and heating water, which is subsidized in Ecuador and is well suited to meet these needs. The batteries will supply the power when a SOC = 44% is available. Below this percentage, although the system will work without complications and the demand will be satisfied, an alarm will be generated only as a reference for the people who are in charge of supervising the electrical system. In reality, the storage system becomes paramount and it is appreciated that the wind turbine will fulfill the role of preventing the battery charge from falling to limits close to zero when demand is maximum.

For the Load Following (LF) strategy, presented in Fig. 14 (b) is established the *SOC* drops to a minimum level, contrary to the case previously analyzed. In this aspect there is a direct dependence on the hybrid system, especially on the PV + HKT power ratio. This option is not exempt from occurring but advantageously in winter there are light water currents but solar radiation that can be reduced at these times or in turn in summer there are high levels of solar radiation but low water flows. In reality, it is possible to cover the electrical power demand of the group of buildings under this configuration of three renewable energy sources operating together.

To finish this part of the energy balance analysis, when the energy generation exceeds expectations when operating together and its production generates excess, it is possible to maintain the batteries at high storage levels.

## 6 Conclusions

The studied solution involves a network of innovative buildings outside the city environment and the daily routine and having 100% clean energy electrical service to coexist in harmony with the exuberant nature. These applications that include renewable energies become attractive for investors, which, in addition to being technically feasible, become economically an interesting business option. In addition, the noise of typical fuel-powered generators is avoided, as well as the smell of fuel that causes severe headaches and discomfort in many people when eating. The transcendental of these renewable energy generation systems is that they can take advantage of the energy potential that the same place has, although in this case we analyze the hybrid system mentioned in other territories, it is possible that there are even other sources that are also well used for the comfort of facilities such as those addressed in this study. The investigation showed the combination (PV/HKT/WT) is from the technical point of view the most advisable to guarantee the electric service to the group of buildings, with the incorporation of an energy storage system. An analysis of the possible configurations shows that the best hybrid solution is the PV/HKT/WT with a SOC exceeding 39% load. It is easily identifiable that the more generation sources are incorporated, the costs tend to increase and therefore the value of the service provided by the concept of electricity will be more expensive, however it is possible to maintain measured values that do not directly affect and in high proportion to the client. Projecting this investment at 25 years with an NPC of less than $37,600 and a COE of $0.386/kWh was obtained.

Additionally, it was identified in the study that only the PV system has an NPC of $16,920 for a SOC of 40% and that it leads to an increase in the NPC to $20,680 if it is desired to reach up to 70%. Then, when HKT/PV are combined, the NPC is $26,320 and their SOC levels are managed to stay between 20% and 60%. The most effective mixture is PV/HKT/WT from an eminently technological analysis, but when determining its NPC it is around $37,600 and it would reach up to 70% SOC.

Given the importance and the guarantee of service that these special infrastructures require, it is recommended to adopt this lasted technological combination because the service rates are high and the expected compensation is a good service, that includes electricity availability.

As of the entry into force of the Creating Opportunities Law promoted by the national government, it is possible to access large discounts on equipment and especially when projects such as the one that was intensely studied are available. According to the incentives, this study can achieve up to $ 20,680 discount for investment volume. Additionally, there are NGOs that can reward these initiatives with economic sums that would motivate investors much more. In this sense, national policies in the coming years will play an important role in the economy in favor of the environment. The proposed system is studied in Ecuador but the methodology and solution can be adapted for any other location and therefore proposes a general design that can be used worldwide.

In this case, the best configuration of the hybrid system was analyzed to meet the demand realistically according to the available energy potential. In techno-economic analysis, it goes one step beyond the mere fact of supplying autonomous energy, it allows us to have a broad panorama for decision-making according to economic availability.

## Author contribution statement

Daniel Icaza: Conceived and designed the experiments; Performed the experiments; Wrote the paper.

David Borge-Diez: Analyzed and interpreted the data; Contributed reagents, materials, analysis tools or data.

## Funding statement

This research did not receive any specific grant from funding agencies in the public, commercial, or not-for-profit sectors.

## Data availability statement

Data included in article/supp. Material/referenced in article.

## Declaration of interest's statement

The authors declare that they have no known competing financial interests or personal relationships that could have appeared to influence the work reported in this paper.

## Figures and Tables

**Fig. 15:**
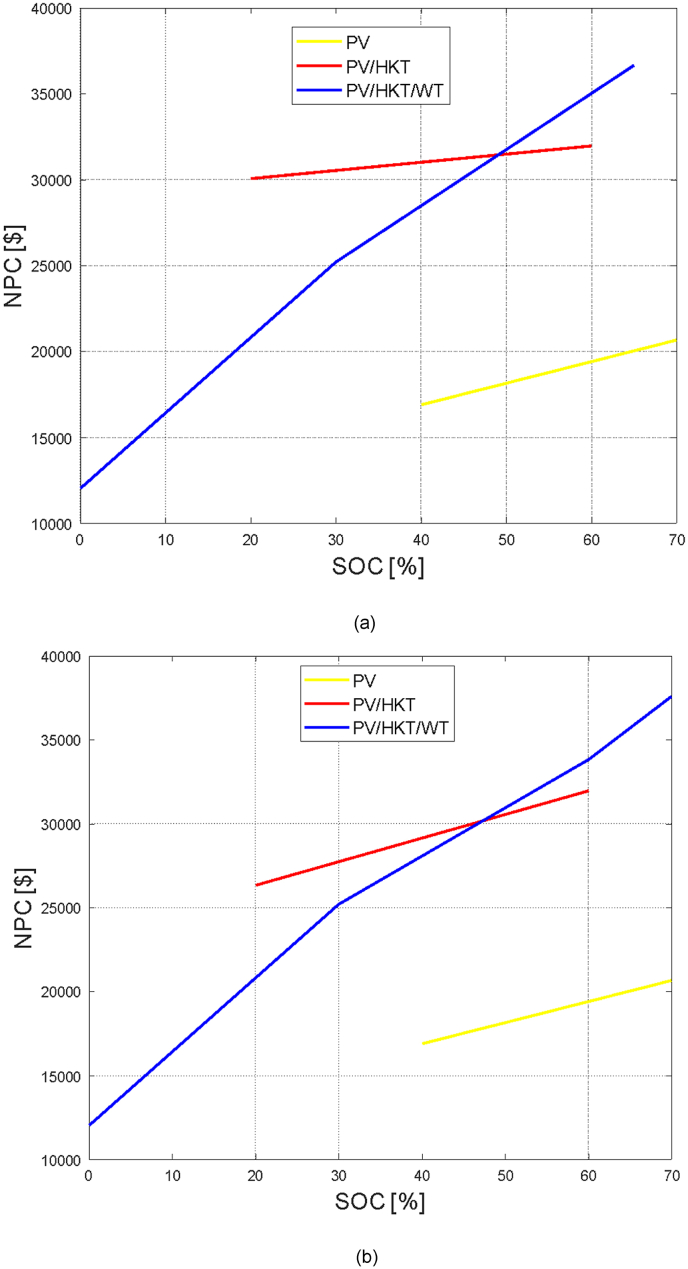
(a) SOC levels of the batteries in relation to the NPC, considering CL. (b) SOC levels of the batteries in relation to the NPC considering LF.

**Fig. 16:**
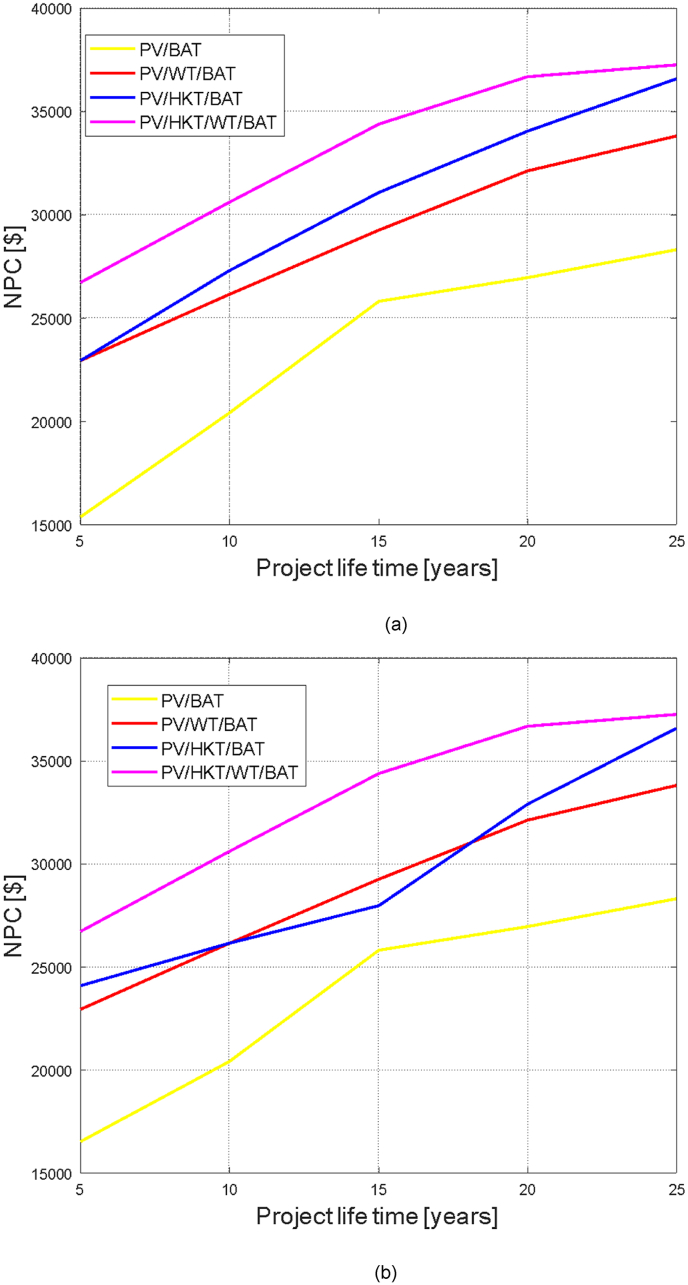
(a) Projection of the hybrid system in relation to the NPC with CL. (b) Projection of the hybrid system in relation to the NPC with LF.

## Appendix

The state of the minimum charge cycle of the battery bank is associated with the NPCs according to the aggregation of the PV, PV/HKT, PV/HKT/WT technologies, as shown in Fig. 15(a). In the case presented in Fig. 15(b), there is an increase in the SOC with the purpose of maintaining higher storage levels for possible load increases that are not very common in normalized load profiles, resulting in a more representative cost due to because there is a greater investment in equipment to reinforce the hybrid system. It has been identified that the percentages of generation of WT and HKT are necessary to maintain SOC levels and do not depend directly on PV since, despite efforts to include more panels, solar radiation is present in 12 h exclusively.

The relationship between NPC and SOC are directly associated, it means a low NPC and a low SOC. If these levels fall below 25% of SOC, there is a risk of shortages in the buildings under study, therefore a SOC above 40% is recommended in balance with the investment, but without putting the electrical service at risk. It is necessary that the resources installed on the site are in the best possible conditions, this implies solar panels that are free of shadows from trees, installation heights of the appropriate wind turbine over 10 m and without considerable obstacles and the HKT turbine in the correct point on the river bank, among other aspects. Regularly achieving high levels of SOC is possible if two or all three technologies reach important levels of generation, in reality it is not always possible but it is necessary that at least one of them allows maintaining the charge in the battery bank during the different hours of the day. In night hours they are called to cover the WT and/or HKT levels or both and cover between 60% and 80%. Considering that there are SOC levels beyond 80% may become unrealistic, considering that as time goes by, technologies lose their initial characteristics, including the battery bank itself.

In the analysis, a fairly close relationship is noted in the results achieved using an LF control and an LC control. The notable feature is that by achieving higher power values in WT, the investment would be relevant and basically in this aspect the potential of the existing resource on the site plays and a good installation to capture either the existing wind tunnels on the site or speeds at hill level.

It is important to carry out a sensitivity analysis based on the useful life of the system, which also includes the battery bank. Typically a projection is made over 15 years, in this study a useful life of 25 years is considered. In Fig. 16(a) and (b) the NPC is analyzed and the respective curves are plotted according to the technologies used to form the hybrid system. When using LC and LF control, results with a high degree of similarity are obtained and over the years the NPC progressively increases in the different technologies used. The combination of the three renewable energy generation systems operating with the battery bank is the one that most increases the value of NPC, it is reasonable since the costs of the equipment will have a direct influence. In second place in relation to the high NPC is the one made up of WT/HKT/BAT, comparatively the one with the lowest NPC is the one made up of PV/BAT, however from the point of view of guaranteeing the service it will be the one with the least confidence introduce.
